# EphA2 overexpression reduces H_2_O_2_-induced damage of lens epithelial cells

**DOI:** 10.1590/1678-4685-GMB-2020-0414

**Published:** 2021-08-06

**Authors:** Qingshan Ji, Jing Liu, Guifang Wang, Lian Liu, Jingxiang Zhong

**Affiliations:** 1 University of Science and Technology of China, Affiliated First Hospital of USTC, Division of Life Sciences and Medicine, Department of Ophthalmology, Hefei, China.; 2 Ophthalmic Hospital of Wuhu, Department of Ophthalmology, Wuhu, China.; 3 Loudi Central Hospital of Hunan, Department of Ophthalmology, China.; 4 Affiliated First Hospital of Jinan University, Department of Ophthalmology, Guangzhou, China.

**Keywords:** EphA2, cell viability, apoptosis, ROS, age-related cataract

## Abstract

Age-related cataract (ARC) is a progressive lens opacification that occurs from middle to old age. Eph-receptor tyrosinekinase-type A2 (EphA2) has been reported to be associated with ARC. This work aims to investigate the molecular mechanism of EphA2 in ARC. We treated human lens epithelial cells (SRA01/04) with different concentration of H_2_O_2_ to induce lens epithelial cell damage. Then, we found that H_2_O_2_ treatment significantly suppressed cell viability and enhanced the expression of EphA2 in the SRA01/04 cells. H_2_O_2_ treatment repressed cell viability and enhanced the levels of reactive oxygen species (ROS) in SRA01/04 cells, which was partly abolished by EphA2 up-regulation. Moreover, EphA2 overexpression reduced H_2_O_2_-induced apoptosis of SRA01/04 cells. EphA2 up-regulation caused an up-regulation of Bcl-2, and repressed the expression of Bax and Cleaved-caspase-3 in the SRA01/04 cells following H_2_O_2_ treatment. In conclusion, our data confirm that EphA2 overexpression enhances cell viability and inhibits apoptosis in the H_2_O_2_-treated SRA01/04 cells, thereby reducing H_2_O_2_-induced damage of lens epithelial cells. Thus, this work provides new insights into the mechanism of EphA2 in ARC.

## Introduction

Age-related cataract (ARC), also known as senile cataract, is a progressive lens opacification that occurs from middle to old age. Surgery is the only effective way to improve the visual function of ARC patients. However, the high cost limits the application of surgical treatment in underdeveloped areas (Ono et al., 2010). At present, cataract is still the main cause of low vision and blindness in the world (Congdon et al., 2003).

The occurrence and development of ARC is a very complicated process. Until now, the specific pathogenesis of cataract has not yet been fully elucidated. Lens epithelial cell apoptosis caused by oxidative stress is considered to be one of the main mechanisms of ARC (Žorić et al., 2015). Oxidative stress leads to disorders of cell metabolism, damages intracellular proteins, DNA and mitochondria, thereby causing cell apoptosis (Fukagawa et al., 2000, Rösen et al., 2001). Lens epithelial cells lack self-renewal ability, so the lens is susceptible to oxidative stress. H_2_O_2_ is used to treat human lens epithelial cells to mimic cataract *in vitro* experiments. H_2_O_2_ treatment increases the apoptotic cells in lens epithelial cells, ROCK1 up-regulation modulates lens epithelial cell apoptosis by regulating p53 expression (Hu et al., 2020). Inhibition of OIP5-AS1 reduces H_2_O_2_-induced B3 cell apoptosis and alleviates lens opacity in the ex vivo cataract model (Jing et al., 2020). 

A previous study has discovered a gene closely associated with the pathogenesis of ARC, namely Eph-receptor tyrosinekinase-type A2 (EphA2) (Jun et al., 2009). EphA2 is widely expressed in corneal tissues, retina tissues and lens. EphA2 plays a crucial role in maintaining the lens transparency. The study revealed that the activated EphA2 suppresses corneal epithelial cell migration through regulating the PI3K-Akt signaling pathway (Kaplan et al., 2012). Cheng et al. (2013) have found that EphA2 participates in maintaining the morphology, structure and differentiation of lens epithelial cells. Knockout of EphA2 destroys the connecting fulcrum between the hexagonal lens epithelial cells in the mouse equator, thereby affecting its structure, proliferation, migration and differentiation of lens epithelial cells. Thus, these data demonstrated the crucial role of EphA2 in the pathogenesis of ARC. However, the mechanism of EphA2 in ARC is still unclear. In brain tissues of EphA2^-/-^ mice, the levels of the pro-apoptotic proteins, Cleaved-caspase-3 and Bax, are significantly decreased, and the levels of the anti-apoptotic protein, Bcl-2, are notably enhanced (Thundyil et al., 2013). EphA2 silencing accelerates apoptosis in mesothelioma cells through enhancing the expression of Bax and Cleaved-caspase-3 (Mohammed et al., 2011). Therefore, we speculated that EphA2 can have a protective effect on lens epithelial cell apoptosis induced by oxidative stress. In this work, we further verified our hypothesis through *in vitro* experiments.

## Material and Methods

### Cell culture

Human lens epithelial cells (SRA01/04 cells) were obtained from Zhongshan Ophthalmic Centre, Sun Yat-sen University (Guangzhou, China). SRA01/04 cells were cultured in Dulbecco’s modified eagle medium (DMEM) (Gibco, Carlsbad, CA, USA) at 37 °C and 5% CO_2_. The medium contained 10% fetal bovine serum (FBS) (Gibco) and 1% penicillin/streptomycin. SRA01/04 cells were treated with different concentration of H_2_O_2_ (50, 100, 200, 300 μM) for 24 h.

### Cell transfection

Full length EphA2 was subcloned into the vector pIRES2-ZsGreen1 (Clontech, Takara, Tokyo, Japan) by a routine method, generating the vector pIRES2-ZsGreen1-EphA2. The empty pIRES2-ZsGreen1-vector was used as control. SRA01/04 cells were seeded into 6-well-plate and incubated for 24 h. Then, SRA01/04 cells were transfected with 2 μg pIRES2-ZsGreen1-vector or pIRES2-ZsGreen1-EphA2 using 5 μL Lipofectamine^TM^ LTX reagent (Invitrogen, Carlsbad, CA, USA) as the instruction described. After 48 h of transfection, the modified SRA01/04 cells were collected and stored at -20 °C for further use. All protocols were performed according to the Declaration of Helsinki and authorized by the Ethics Committee of the First Affiliated Hospital of USTC.

### Quantitative real-time PCR (qRT-PCR)

QRT-PCR was used to measure EphA2 expression in SRA01/04 cells. TRIzol reagent (Invitrogen) was used to extract total RNA from SRA01/04 cells. RNA integrity was examined by 1.5% agarose gel electrophoresis. Complementary DNA was generated using First Strand cDNA Synthesis Kit (TOYOBO, Tokyo, Japan). PCR was performed using SYBR qPCR Mix (TOYOBO) on a Real-time PCR system (Bio-Rad, Hercules, CA, USA). GAPDH was used as an internal control. Primer sequences were as follows: EphA2-F: 5’-CCACCACAACATCATCCGCCTA-3’; EphA2-R: 5’-CACGCTGAACTCGCCATCCTT-3’; GAPDH-F: 5’-GACACCCACTCCTCCACCTTT-3’; GAPDH-R: 5’-CCACCCTGTTGCTGTAGCCAA-3’. The relative expression of EphA2 was analyzed using 2^-∆∆CT^ method for quantification.

### Western blot (WB) analysis

SRA01/04 cells were lysed using RIPA Lysis Buffer (Beyotime Biotechnology, Shanghai, China) to extract total protein from cells. BCA Protein Assay Kit (Beyotime Biotechnology) was used to measure the concentration of proteins. Protein samples were separated by 10% SDS-PAGE, and the separated proteins were transferred onto PVDF membranes (Merck Millipore, Billerica, MA, USA). The membranes were blocked with blocking buffer (Beyotime Biotechnology) and then incubated with primary antibodies, anti-Bcl-2 (1: 1000, ProteinTech Group, Chicago, IL, USA), anti-Bax (1: 1000, ProteinTech Group), anti-Caspase-3 (1: 1000, ProteinTech Group) or anti-Cleaved-caspase-3 (1:500, Abcam, Cambridge, MA, USA) at 4 °C overnight. The membranes were incubated with goat anti-rabbit IgG-labeled with horseradish peroxidase (1:1000, Beyotime Biotechnology). GADPH antibody (1:1000, Goodhere, Hangzhou, China) was used as a reference protein for normalization. The data were analyzed by Image J software.

### Cell viability

Viability of SRA01/04 cells was examined by the Cell Counting Kit-8 (BestBio, Shanghai, China). SRA01/04 cells were seeded into 96-well plate and incubated with different concentrations of H_2_O_2_ for 24 h. Then, 10 μL CCK-8 reagent was added into each well, and incubated with cells for 2 h. The absorbance of each well at 450 nm was measured on a microplate reader (Tecan Safire, Männedorf, Switzerland).

### Apoptosis

SRA01/04 cells were treated with 200 μM H_2_O_2_ for 24 h, and then the apoptosis of SRA01/04 cells was examined applying Annexin V-FITC/PI Apoptosis Detection Kit (eBioscience, San Diego, CA, USA). Briefly, SRA01/04 cells were washed with PBS buffer, and then stained with Annexin V-FITC (10 μL) and PI Staining Solution (10 μL) at darkness for 20 min. Then, the cell suspension was mixed with 400 μL 1 × Binding Buffer on ice. Apoptosis was determined by flow cytometry within 1 h.

### Detection of reactive oxygen species (ROS)

Reactive Oxygen Species Assay Kit (KeyGen Biotech, Nanjing, Jiangsu) was used to measure the levels of ROS in SRA01/04 cells following the protocol of the manufacturer. SRA01/04 cells (2 × 10^5^ cells/well) were incubated with 200 μM H_2_O_2_ in a 6-well plate for 24 h. After that, SRA01/04 cells were stained with 10 μM DCFH-DA at darkness for 25 min. The fluorescence intensity of the SRA01/04 cells was observed under a confocal laser scanning microscope.

### Statistical analysis

The independent experiments were performed three times, and each independent experiment contained three technical replicates. The data were analyzed using SPSS 16.0 (IBM, Armonk, NY, USA) and GraphPad Prism 5 (San Diego, CA, USA) statistical software. The values were reported as mean ± standard deviation. Two-tailed Student’s t-tests were used to analyze the statistical difference between two groups. One-way ANOVA or Least-significant difference was used to analyze the statistical difference among multiple groups. *P* < 0.05 was considered as a significant difference.

## Results

### H_2_O_2_ treatment reduced cell viability and enhanced EphA2 expression in SRA01/04 cells.

**Figure 1 -:**
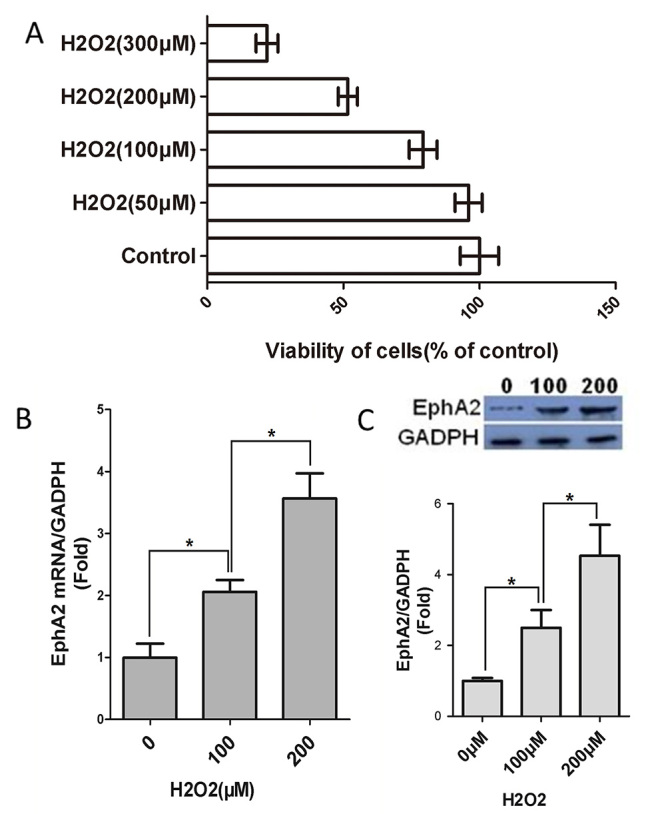
H_2_O_2_ repressed cell viability and enhanced EphA2 expression in SRA01/04 cells. SRA01/04 cells were treated with different concentration of H_2_O_2_ (50, 100, 200, 300 μM) for 24 h. (A) CCK-8 assay was performed to examine cell viability of SRA01/04 cells. (B-C) QRT-PCR and WB were performed to examine the gene and protein expression of EphA2 in SRA01/04 cells. ^*^ *P* < 0.05, vs. Control or 0 or 100 group.

To explore the biological role of EphA2 in ARC, SRA01/04 cells were treated with H_2_O_2_ to mimic cataract *in vitro*. The data obtained from CCK-8 assay showed that 50 μM H_2_O_2_ had no effect on SRA01/04 cell viability. However, H_2_O_2_ at 100, 200 and 300 μM reduced SRA01/04 cell viability, although at different extent (Figure 1A). Thus, we suggested that H_2_O_2_ repressed viability of SRA01/04 cells in a dose-dependent manner. Subsequently, we found that the gene and protein expression of EphA2 in SRA01/04 cells were significantly enhanced in the presence of 100 and 200 μM H_2_O_2_, especially 200 μM of H_2_O_2_ (Figure 1B-C). These data revealed that H_2_O_2_ treatment reduced cell viability and enhanced EphA2 expression in SRA01/04 cells.

### EphA2 overexpression promoted cell viability of H_2_O_2_-treated SRA01/04 cells.

**Figure 2 -:**
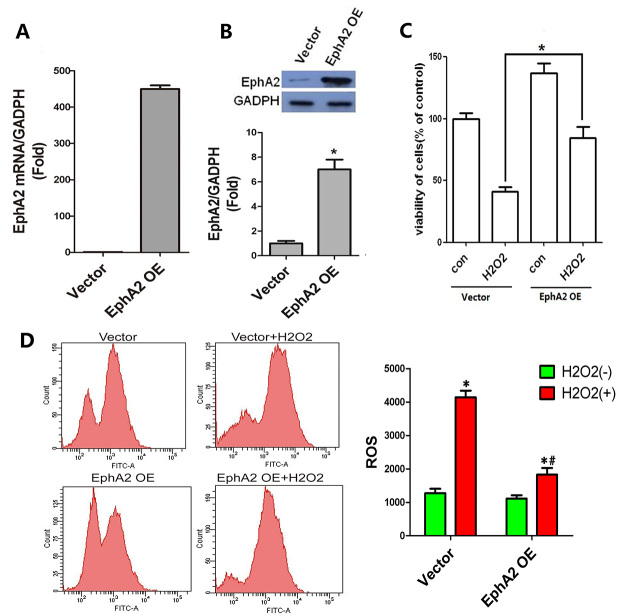
EphA2 overexpression enhanced cell viability and reduced ROS levels in H_2_O_2_-treated SRA01/04 cells. SRA01/04 cells were transfected with pIRES2-ZsGreen1-EphA2 or pIRES2-ZsGreen1-vector, and then treated with 200 μM H_2_O_2_ for 24 h. (A-B) QRT-PCR and WB were performed to examine the gene and protein expression of SRA01/04 cells. (C) CCK-8 assay was performed to detect cell viability of SRA01/04 cells. (D) The levels of ROS in SRA01/04 cells were assessed using DCFH-DA- fluorescent probe. ^*^ *P* < 0.05, vs. Vector group; ^#^ *P* < 0.05, vs. H_2_O_2_ group.

Next, EphA2 was overexpressed in SRA01/04 cells to verify the biological role of EphA2 in H_2_O_2_-treated SRA01/04 cells. QRT-PCR and WB data showed that the gene and protein expression of EphA2 in SRA01/04 cells were significantly enhanced in the presence of pIRES2-ZsGreen1-EphA2 (Figure 2A-B). We also found that EphA2 overexpression promoted cell viability of SRA01/04 cells. The cell viability of SRA01/04 cells was notably reduced following treatment of H_2_O_2_, which was enhanced by EphA2 overexpression (Figure 2C). Moreover, compared with SRA01/04 cells, the levels of ROS were enhanced by H_2_O_2_ treatment. EphA2 up-regulation severely reduced the levels of ROS in H_2_O_2_-treated SRA01/04 cells (Figure 2D). Therefore, these findings revealed that EphA2 overexpression promoted cell viability and reduced the levels of ROS in H_2_O_2_-treated SRA01/04 cells.

### EphA2 overexpression repressed apoptosis of H_2_O_2_-treated SRA01/04 cells

**Figure 3 -:**
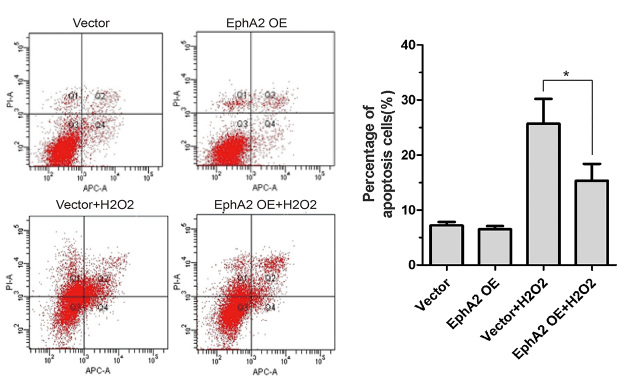
EphA2 overexpression repressed apoptosis of H_2_O_2_-treated SRA01/04 cells. SRA01/04 cells were transfected with pIRES2-ZsGreen1-EphA2 or pIRES2-ZsGreen1-vector, and then treated with 200 μM H_2_O_2_ for 24 h. Flow cytometry was performed to estimate apoptosis of SRA01/04 cells. ^*^ *P* < 0.05, vs. Vector + H_2_O_2_ group.

**Figure 4 -:**
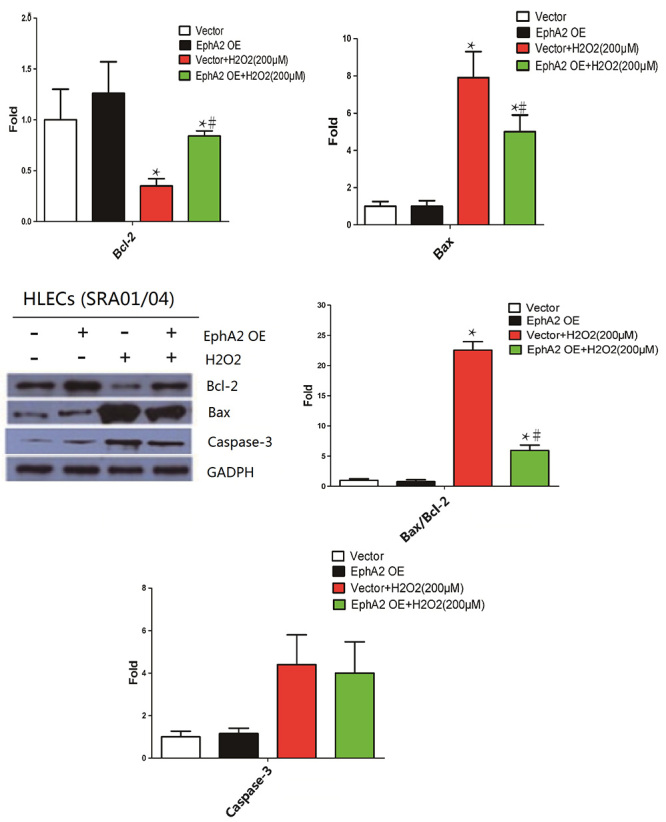
EphA2 overexpression enhanced Bcl-2 expression and inhibited Bax expression in H_2_O_2_-treated SRA01/04 cells. SRA01/04 cells were transfected with pIRES2-ZsGreen1-EphA2 or pIRES2-ZsGreen1-vector, and then treated with 200 μM H_2_O_2_ for 24 h. WB was performed to examine the expression of Bcl-2, Bax, Caspase-3 and the ratio of Bax/Bcl-2 in SRA01/04 cells. ^*^ *P* < 0.05, vs. Vector group. ^#^ *P* < 0.05, vs. Vector + H_2_O_2_ group.

We further explored the effect of EphA2 overexpression on apoptosis of H_2_O_2_-treated SRA01/04 cells by flow cytometry. Figure 3 shows that EphA2 overexpression had no effect on apoptosis of SRA01/04 cells. However, EphA2 overexpression led to a decrease in apoptosis of SRA01/04 cells in the presence of H_2_O_2_ (Figure 3). Compared with normal SRA01/04 cells, H_2_O_2_ treatment notably reduced Bcl-2 expression and enhanced Bax expression in SRA01/04 cells. EphA2 overexpression caused an up-regulation of Bcl-2 and a down-regulation of Bax in H_2_O_2_-treated SRA01/04 cells (Figure 4). Thus, H_2_O_2_ treatment enhanced the expression of Bcl-2/Bax, which was abolished by EphA2 overexpression. Furthermore, the expression of Caspase-3 in SRA01/04 cells was enhanced in the presence of H_2_O_2_. EphA2 overexpression had on influence on the expression of Caspase-3 in SRA01/04 cells (Figure 4). However, H_2_O_2_ treatment significantly enhanced Cleaved-caspase-3 expression in SRA01/04 cells. EphA2 overexpression led to a decrease of Cleaved-caspase-3 expression in SRA01/04 cells (Figure S1). Taken together, these data confirmed that EphA2 overexpression repressed apoptosis of H_2_O_2_-treated SRA01/04 cells.

## Discussion

The oxidation-antioxidant system plays a vital role in maintaining the normal physiological metabolism of the lens and the stability of the internal environment (Truscott, 2005). H_2_O_2_ is one of the main oxides in the lens. Exposure of H_2_O_2_ in lenses causes rupture of the lens capsule, degenerative lens epithelial cells and lens opacity, which is effectively prevented by Ginsenoside Rg1 (Zhang et al., 2021). In the present study, we also treated SRA01/04 cells with H_2_O_2_ to induce lens epithelial cell damage. We found that H_2_O_2_ notably repressed cell viability of SRA01/04 cells in a dose-dependent manner. In addition, EphA2 was highly expressed in SRA01/04 cells after treated with different concentration of H_2_O_2_. EphA2 plays an indispensable role in the cytoarchitecture and refractive quality of the lens, and it is important in maintaining lens clarity with age (Jun et al., 2009; Shi et al., 2012). Thus, these data suggested that EphA2 was closely associated with H_2_O_2_-induced lens epithelial cell damage.

Accumulating research has confirmed the biological role of EphA2 in ARC. EphA2 has been closely associated with loss of eye lens transparency, or cataract. Epha2 participates in the complex, global patterning of lens fiber cells, which is necessary for maximal optical quality (Zhou and Shiels, 2018). Common variants in EPHA2 showed significant association with cortical cataract, such as rs7543472 and rs3754334 (Yang et al., 2013, Aslam et al., 2020). Previous studies mostly focused on the single nucleotide polymorphism of EphA2 in ARC. In the present study, we attempted to determine the specific mechanism of action of EphA2 in ARC. We found that EphA2 overexpression enhanced cell viability of H_2_O_2_-treated SRA01/04 cells. In addition, H_2_O_2_ treatment caused an increase in the levels of ROS in SRA01/04 cells, which was effectively suppressed by EphA2 overexpression. H_2_O_2_ is a powerful ROS that can penetrate the cell membrane, damage the mitochondrial respiratory chain and DNA, and induce cell apoptosis and damage (Silvers and Bowden, 2010). Oxidative stress-induced lens epithelial cell injury plays an important role in the pathogenesis of ARC (Lu et al., 2019). Our data indicated that EphA2 overexpression may reduce H_2_O_2_-induced SRA01/04 cell damage in the development of ARC.

EphA2 up-regulation had an inhibiting effect on apoptosis of H_2_O_2_-treated SRA01/04 cells. Moreover, EphA2 overexpression caused an up-regulation of Bcl-2, and led to a down-regulation of Bax in the H_2_O_2_-treated SRA01/04 cells. EphA2 overexpression had no effect on the expression of Caspase-3 in the H_2_O_2_-treated SRA01/04 cells. However, EphA2 overexpression led to a decrease of Cleaved-caspase-3 expression in H_2_O_2_-treated SRA01/04 cells. Bcl-2 is a mitochondrial membrane protein, which plays a regulatory role in cell apoptosis. Bcl-2 inhibits the translocation of cytochrome C, blocks the activation of Caspase, and then reduces cell apoptosis (Czabotar et al., 2014). Bcl-2 up-regulation or Bax down-regulation reduces H_2_O_2_-induced cell apoptosis (Fang et al., 2012). Thus, EphA2 regulates H_2_O_2_-induced apoptosis of SRA01/04 cells through regulating the expression of apoptosis-related proteins, Bcl-2, Bax and Cleaved-caspase-3. Taken together, these findings demonstrated that EphA2 overexpression attenuated H_2_O_2_-induced damage of lens epithelial cells through enhancing cell viability and inhibiting apoptosis (Figure S2).

A previous study has demonstrated that inhibition of NEAT1 attenuates H_2_O_2_-induced oxidative stress and apoptosis of SRA01/04 cells via NF-κB/p65 and p38 MAPK signaling pathways (Zhou et al., 2020). EphA2 down-regulation represses the activation of MAPK and AKT signaling pathways and extracellular matrix in lens cells, and then induces the occurrence of cataract (Ma et al., 2017). Thus, we speculated that EphA2 overexpression may repress H_2_O_2_-induced oxidative stress and apoptosis of SRA01/04 cells through MAPK signaling pathway. However, the underlying mechanism of EphA2 in alleviating the development of ARC still needs further research. 

In conclusion, our data revealed that EphA2 overexpression enhanced cell viability and inhibited apoptosis in the H_2_O_2_-treated SRA01/04 cells, thereby reducing H_2_O_2_-induced damage of lens epithelial cells. Thus, this work provides new insights into the mechanism of EphA2 in ARC.

## Supplementary material

The following online material is available for this article:

Figure S1 - EphA2 overexpression enhanced Cleaved-caspase-3 expression in H_2_O_2_-treated SRA01/04 cells.

Figure S2 - The mechanism of action of EphA2 in ARC.
