# Supplementary material for: EphA2 overexpression reduces H2O2-induced damage of lens epithelial cells
Source: Genet Mol Biol. 2021 Aug 6;44(3):e20200414. doi: 10.1590/1678-4685-GMB-2020-0414 (PMC8345112; doi:10.1590/1678-4685-GMB-2020-0414)
Supplement: Figure S2 - [file 1415-4757-GMB-44-3-e20200414-s2.pdf]

Genetics and Molecular Biology
https://doi.org/10.1590/1678-4685-GMB-2020-0414

Supplementary Material to "EphA2 overexpression reduces H2O2-induced damage of lens epithelial cells"

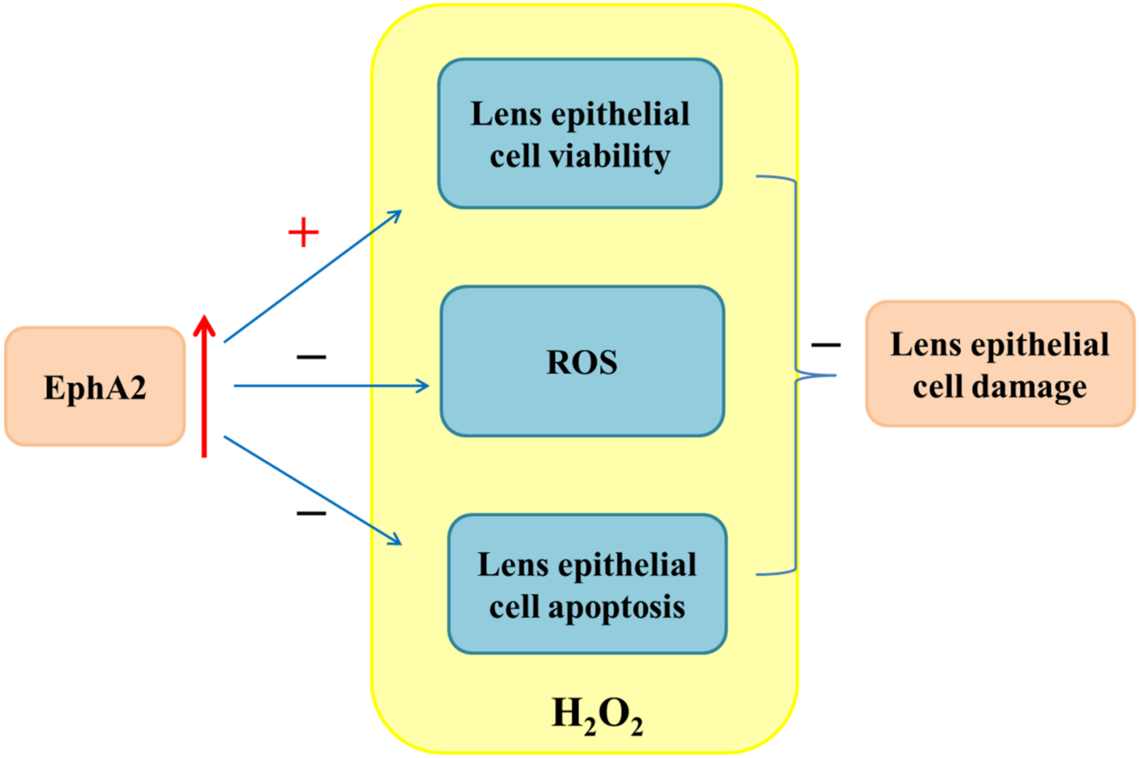

Figure S2 - The mechanism of action of EphA2 in ARC.
